# HIV- and hepatitis C-related risk behaviors among people who inject drugs in Uganda: implications for policy and programming

**DOI:** 10.1186/s12954-019-0324-4

**Published:** 2019-09-03

**Authors:** Matayo Baluku, Twaibu Wamala, Denis Muhangi

**Affiliations:** 1 Infectious Diseases Institute, College of Health Sciences, Makerere University, Kampala, Uganda; 2 Uganda Harm Reduction Network, Kampala, Uganda; 3 School of Social Sciences, Makerere University, Kampala, Uganda

**Keywords:** People who inject drugs, Injecting drug use, Hepatitis C virus, HIV, Risk behavior, Heroin, Female sex workers, Harm reduction, Uganda

## Abstract

**Background:**

There is a dearth of evidence on injecting drug use and associated HIV and hepatitis C virus (HCV) infections in Uganda. As such, policy and programming for people who inject drugs (PWID) is limited due to scarcity of epidemiological data. We therefore conducted this study to assess the injecting drug and sexual practices among PWID in Kampala Capital City and Mbale Municipality.

**Methods:**

Using a rapid situation assessment framework, we conducted semi-structured interviews among 125 PWID (102 males and 23 females)—recruited through outreach and snowball sampling. We assessed their injecting drug and sexual practices. We also conducted 12 focus group discussions among PWID and 30 in-depth interviews among key informants.

**Results:**

A total of 125 PWID (81.6% males and 18.4% females) were recruited into the study. Approximately three quarters of PWID started injecting before the age of 25. More females (21.7%) compared to males (13.7%) started injecting by the age of 17. Fifty-seven percent of the PWID in Kampala and 50% in Mbale shared injecting equipment in the last 3 months prior to the study. There was an emerging practice of mixing drugs with blood and sharing it among different PWID as a sign of oneness. Heroin was being injected by 72% of the participants. Less than one half of the PWID had used a condom during the last casual sex, and 42.7% did not use a condom the last time they engaged in sex work. Seventy-six percent of the PWID had undertaken an HIV test in the last 12 months, and 9.2% self-reported to be HIV positive.

**Conclusions:**

This study highlights the need for introducing harm reduction policies and services including increased access to sterile injecting equipment and education around safer injecting and sexual practices. Programs for PWID should also address the specific needs of female sex workers who inject drugs.

## Introduction

Globally, an estimated 275 million people used drugs at least once in 2016, 10.9 million of whom injected their drugs [1]. Injecting drugs carries a high risk of human immunodeficiency virus (HIV) and viral hepatitis transmission if sterile injecting equipment is not easily accessible and injecting equipment is shared among users. In 2016, more than half of the people who inject drugs (PWID) worldwide were living with hepatitis C virus (HCV); one in eight was living with HIV, and 82.4% were co-infected with both HIV and HCV [2–5]. The risk of acquiring HIV for PWID in 2017 was 22 times higher than that for people who did not inject drugs [6].

Whereas hepatitis B virus (HBV) and HIV are transmitted via blood or body fluids [7], sharing injecting equipment poses the greatest risk of HCV transmission among PWID [8]. Also, whereas there is no increased risk of HCV transmission in a long-term heterosexual relationship, the risk of transmission increases with multiple sexual partners and among women who are infected with HIV or other sexually transmitted diseases [9]. Similarly, engaging in sex work, younger age, cocaine injecting, depression, requiring help injecting, having unsafe sex with a regular partner, and having an HIV-positive sexual partner are associated with HIV infection among PWID [10–12].

Risk factors for HCV among PWID include sharing needles and other injecting equipment [13, 14], longer duration of injecting career [15], increased frequency of injection [15, 16], requiring help injecting [17, 18], being female [15, 19], and history of imprisonment [15, 16].

Despite the heavy burden of injecting drug use and its associated risks, and burden of HIV and HCV among PWID, effective harm reduction interventions that can help prevent their spread are severely lacking in many countries including Uganda [20–22]. In Sub-Saharan Africa, only eight countries operate the needle and syringe program (NSP); seven offer some form of opioid substitution therapy (OST), and ten have explicit support for harm reduction contained in national policy documents [21]. The current policies and guidelines for prevention and treatment of HIV and AIDS in Uganda do not support OST and NSP [23].

While Uganda is one of the 179 countries where injecting drug use is present [20, 21], data on PWID is limited. No studies have enquired about risk injecting and sexual practices for HIV and HCV among PWID. We therefore conducted this study to assess the risk practices among PWID in Uganda. We compared PWID sampled from Kampala, the only capital and central business city of Uganda, and Mbale Municipality, an urban center in Eastern Uganda.

## Methods

This rapid situation assessment (RSA) [24]—conducted from February to September, 2017—was part of a broader study to establish the current situation of injecting drug use in Uganda and identify the country-specific links between injecting drug use and HIV and HCV risk. It sought to generate evidence on “what practices and behaviors increase HIV and HCV risk among PWID in Kampala and Mbale?”

The study was conducted in two different settings: Kampala Capital City and Mbale Municipality. Kampala the only capital and business city of Uganda was purposively selected because it is thought to have the biggest number of people who inject drugs in Uganda. Mbale on the other hand is one of the major towns in Eastern Uganda and a transit route from neighboring Kenya. It is a blossoming urban center [25].

An inductive approach [26, 27] was used for this rapid assessment, whereby initial data collected informed planning and decisions about subsequent data collection. The rationale for this approach was to allow the research team to make sense of the data collected, determine its utility and adequacy, identify gaps, and then collect additional data if necessary.

Participants of this study were required to be PWID, both male and female aged 18 years and above; had injected drugs for non-medical purposes at least once in the past 3 months; were residents within the two study sites; and had voluntarily consented to take part in the study. Those who were deemed to be high on drugs at the time of contact were rescheduled to be interviewed at another time, or if that was not possible, they were excluded. The key informants were purposively selected people presumed to have an understanding of PWID in Uganda. They included officials from the Ministry of Health, Butabika National Mental Referral Hospital, and health workers from mental health units; officers and men of the narcotics control division of Uganda Police Force and Uganda Prisons Services; and staff of civil society organizations working with PWID.

A total of 125 PWID (95 in Kampala and 30 in Mbale) recruited through snowballing and intercepts initiated by outreach workers in known drug-use areas (hotspots) participated in the study. Data was collected from them using semi-structured interviews.

Thirty in-depth interviews (IDIs) among key informants and 12 focus group discussions (FGDs) among PWID were also conducted. Seven FGDs were done in Kampala and five in Mbale. They were conducted mainly within the hotspots. Observations of the settings and environments in which injecting drug use took place were done. The research team took extensive handwritten notes during data collection. Only in a few cases was audio-recording done.

The survey collected information on sociodemographic data, drug use patterns, risk perceptions, and service utilization from PWID. The FGDs among PWID were intended to generate in-depth information about drug use behaviors and patterns, perceptions of risk, access to and utilization of HIV and harm reduction services, shared norms and meanings, and transition from non-injecting to injecting drug use. Similarly, IDIs provided information about the locations (hotspots) where injecting drug use takes place, the context of drug use, the types of drugs used, the services available and the extent to which PWID utilized them, and the links between drug use and other risk behaviors and health harms. Other outcomes for the same study are described by Platt et al. [28]. The PWID also participated in the population size estimates described elsewhere [29].

The quantitative data collected was analyzed using MS Excel and described in terms of frequencies and percentages on the different variables. We adopted the thematic analysis approach described by Braun and Clark [30] to analyze the qualitative data progressively as it was collected. It generally followed the steps outlined: organizing and ordering the raw data; coding the interviews; listing and sorting the data; categorizing and summarizing the data; interpreting the data; and triangulating, validating, and drawing conclusions. The information was classified according to themes and domains and presented in form of taxonomies that reveal emerging patterns.

## Results

### Demographic characteristics of the sample

A total of 102 (81.6%) males and 23 females (18.4%) PWID were recruited into the study. The mean age was 27.4 (females, 25.9; males, 27.8). Only 30.4% of the participants were married or living together. At least 71 (56.8%) participants had attained a minimum of secondary education, and 17 (13.6%) earned their living through sex work. Table 1 has details of the demographic characteristics of the participants, stratified by study area.

**Table 1:** Demographic characteristics of the participants

Characteristic	Kampala	Mbale
	Number (%)	Number (%)
Biological sex of participant		
Male	77 (81.1)	25 (83.3)
Female	18 (18.9)	5 (16.7)
Age of participant		
18–24	32 (33.7)	12 (40.0)
25–29	35 (36.8)	16 (36.7)
30–34	15 (15.8)	3 (10.0)
35 and above	13 (13.7)	4 (13.3)
Marital status		
Never married/single	47 (49.5)	17 (56.7)
Married or living together	28 (29.5)	10 (33.3)
Divorced/separated	19 (20.0)	3 (10.0)
Widowed	1 (1.1)	0 (0.0)
Highest level of education		
No education	3 (3.2)	0 (0.0)
Primary incomplete	19 (20.0)	7 (23.3)
Completed primary education	23 (24.2)	2 (6.7)
Secondary	34 (35.8)	16 (53.3)
Post-secondary	16 (16.8)	5 (16.7)
Occupation of respondent		
Artisan/builder/carpenter/mechanic	19 (20.0)	4 (13.3)
Temporary/informal/casual work	9 (9.5)	13 (43.3)
Sex work	15 (15.8)	2 (6.7)
Transporter	15 (15.8)	0 (0.0)
Trade/business	9 (9.5)	2 (6.7)
Entertainer/artist	8 (8.4)	0 (0.0)
Fishing	7 (7.4)	0 (0.0)
Salon/beauty/health club	3 (3.2)	0 (0.0)
Student	0 (0.0)	2 (6.7)
Bar/hotel worker	0 (0.0)	1 (3.3)
Other	4 (4.2)	5 (16.7)
None	6 (6.3)	1 (3.3)

### Injecting drug practices among PWID in Kampala and Mbale

The injecting practices of the respondents are shown in Table 2. At least 15.2% of PWID (13.7% in Kampala and 20% in Mbale) started injecting before the age of 17. Overall, a bigger proportion of females (21.7%) compared to that of males (13.7%) start injecting before the age of 17. By the age of 25, at least 81.6% of the PWID had started injecting.

**Table 2:** Participants’ injecting drug practices

Injecting practices	Kampala	Mbale
	Freq (%)	Freq (%)
Age first injected		
10–17	13 (13.7)	6 (20.0)
18–24	63 (66.3)	20 (66.7)
25+	19 (20.0)	4 (13.3)
Type of drug injected in the past 4 weeks		
Brown heroin	33 (34.7)	23 (76.7)
White heroin	33 (34.7)	5 (16.7)
Cocaine	19 (20.0)	1 (3.3)
Morphine	3 (3.2)	0 (0.0)
Pethidine	0 (0.0)	2 (6.7)
Antiretroviral medications + diazepam	2 (2.1)	2 (6.7)
Other	5 (5.3)	3 (10.0)
Number of times a needle was used before disposal		
1	28 (29.5)	10 (33.3)
2–4	41 (43.2)	15 (50.0)
5–9	12 (12.6)	1 (3.3)
10+	13 (13.7)	1 (3.3)
Do not know/do not remember	1 (1.1)	3 (10.0)
Number of times a syringe was used before disposal		
1	27 (28.4)	6 (20.0)
2–4	36 (37.9)	12 (40.0)
5–9	10 (10.5)	7 (23.3)
10+	20 (21.1)	2 (6.7)
Do not know/do not remember	2 (2.1)	3 (10.0)
Sharing practices		
Injected using needles/syringes used by someone else	57 (60)	15 (50)
Injected using a syringe after someone has squirted into it from their syringe (frontloading/backloading/splitting)	59 (62.1)	13 (43.3)
Used a filter or cotton into which someone else had previously used to draw up drugs with their needle/syringe	43 (45.3)	4 (13.3)
Drew up drug solution into syringe from a mixing container (spoon or glass) from which someone else had previously drawn up	64 (67.4)	15 (50)

Heroin was the most injected drug in both Kampala and Mbale in the last 4 weeks preceding the study. Study participants however kept a distinction between white heroin and brown heroin (commonly referred to as brown sugar). Brown heroin was injected by 76.7% of PWID in Mbale, while in Kampala, the same proportions (34.7%) had injected brown heroin as white heroin. When participants were asked which drug they had injected most frequently in the past, 57.9% in Kampala mentioned brown heroin, compared to 28.4% who mentioned white heroin. We understood that brown heroin/brown sugar was an adulterated or impure form of heroin.

The dominance of brown heroin is attributed to its cheaper cost compared to white heroin, and it could be both smoked and injected. At least 57% of all respondents shared needles and syringes, and only 30.4% used a needle once before disposal. Heroin was injected by 72% of the participants. PWID in both Kampala and Mbale use a concoction of antiretroviral drugs (ARVs) meant for the treatment of AIDS mixed with diazepam—a sedative.

Qualitative results indicated an emerging practice of sharing blood of individuals who are already “high” on drugs or mixing drugs with blood as a sign of oneness as well as wide spread of sharing of injecting equipment:

“we are one blood … one brotherhood, if our colleague is already high, we can draw his blood and inject ourselves to get high too … ” PWID FGD, Bwaise

“ … cannot have this drug and fail to share with my brother here because I know at one time I will not have money and he will share with me so for us here we are all brothers and we are one family” FGD, PWID, Kawempe

“drugs are expensive sometimes we don’t change needles in order to avoid losing any drop of the drug” FGD, MSM PWID, Mbale

### Sexual practices among PWID in Kampala and Mbale

On average, more than a half of the respondents had sex with two or more partners in the month preceding the study. Only 47% had used a condom during the last casual sex, and of the 65 participants who had sold or bought sex, only 54% used a condom. Details of practices are provided in Table 3.

**Table 3:** Participants’ sexual practices

Sexual practice	Kampala	Mbale
	*n* (%)	*n* (%)
Had sex with 2 or more partners in the last 30 days	46 (48.4)	18 (60.0)
Used a condom at the last casual sex	45 (47.4)	14 (46.7)
Used a condom the last she/he bought or sold sex	25 (50%)	10 (66.7)
Ever had sex with someone of the same sex	2 (2.2)	1 (3.3)
Exchange sex for money, drugs, or gifts in the last 6 months	10 (10.5)	4 (13.3)
Had HIV test in the last 12 months	76 (80)	19 (63.3)
Self-reported HIV positive	7 (9.2)	0 (0.0)
Have a non-injecting partner	58 (61.1)	18 (60.0)

## Discussion

This study is the first to report population-based risk behaviors among PWID in Kampala Capital City and Mbale Municipality, Uganda. Our findings indicate high levels of risky injection behaviors. The median age at first injection was 19 (14–25) with 15.2% of the participants having started injecting by the age of 17 and 81.6% before the age of 25. This age was comparable to similar studies conducted in South Africa [31, 32]. Other studies have found out that up to 45% of the PWID began injecting before they were 15 years old [33]. While most studies on PWID exclude young people aged 15–24 especially those under the age of 18 [34], it is important to note that injecting drug use in this age group is important because early onset of injecting, and being a new injector, has been associated with increased risks of HIV and hepatitis C transmission [35]. For some young people, drug use is a part of adolescent experimentation, socialization, risk taking, and reward seeking, particularly in the presence of peers [36]. They are more likely to inject drugs in groups and to share injecting equipment as they might not be able to afford to buy their own [36, 37, 38]. Young people who inject drugs in Uganda, including those below the age of 18, will need special attention for harm reduction programming as well as research.

This study established that three in every five PWID in Kampala and one in two PWID in Mbale share injecting equipment. Sharing of injecting equipment is well studied as a high risk practice for transmission of HIV and viral hepatitis [4, 8, 13]. Limited access to new syringes and needles and limited knowledge on the risks were important contributors to the behaviors. This, in case of Uganda, is worsened by the lack of a policy framework under which to provide such services. Studies have suggested that knowledge gap especially for HCV transmission among PWID also contributed to its high prevalence [39–41]. Similar studies in Nairobi, Kenya, also established that HIV was independently associated with lifetime practice of using previously used needles and syringes and longer time since first injection [42], while one in South Africa [31] did not. They, like others before, highlight the lack of needle and syringe programs and criminalization of PWID as contributing factors to high risk injecting behaviors [32, 43].

Sexual risk behaviors are also significant among PWID in Uganda due to multiple sex partners, irregular use of condoms, and selling of sex. Female participants, like in most studies, were more likely to sell sex than men. Sex work is a common means to support drug use and that of their partners, and sex workers might use drugs to enhance sexual performance and as a coping strategy—to obtain courage to approach clients and stand the cold on the streets. Additionally, use of certain drugs may increase sexual desire or lower behavioral inhibitions, further affecting risk perception [44–46].

The study also established that the self-reported HIV prevalence among PWID in Kampala, at 9.2%, is still higher than the national prevalence of 6.2% [47]. Given that over 60% of the PWID had non-injecting partners, the likelihood of bridging to the general population is high.

### Limitations

Firstly, the study was conducted in only two sites, that is, Kampala and Mbale, even though there is a lack of data on a national scale. Secondly, the study focused on HIV and HCV, leaving out other equally important infections such as tuberculosis and HBV infection. Further, in this study, we did not interview PWID under the age of 18, although they exist, neither did we access the middle/upper class or corporate PWID. Lastly, we did not test blood for HIV and HCV but only relied on self-reported test results. Some studies have suggested that self-reported HIV test results are likely to be inaccurate due to the effects of social desirability bias and post-test seroconversion [48], or when respondents fail to understand the meaning of a positive or negative test result and fail to remember the result [49]. Similarly, routine service utilization data, like most sentinel surveillance data [50], are from non-random samples and might not be representative of the prevalence of HIV and HCV among the PWID.

Despite the above limitations, the results generated by the study still provide reliable and useful insights into the dynamics and risks of injecting drug use in Kampala and Mbale towns.

## Conclusion

Our findings indicate injecting drug use is a growing problem in Kampala Capital City and Mbale Municipality. PWID reuse and share injecting equipment, as well as mix drugs with blood and share the mixture. PWIDs also engage in risky sexual practices including multiple partners, irregular condom use, and sex work. These are high risks for HIV and HCV infection. With over 60% of sexually active PWID having non-injecting sex partners, PWID can potentially fuel the HIV and viral hepatitis epidemic in the general and non-injecting population in Uganda.

### Implications for policy and programming

Given the gravity of the risks of injecting drug use in Uganda and lack of comprehensive interventions for PWID, the Ministry of Health should embrace harm reduction—including OST and NSP, as the overarching strategy to respond to drug injecting-related risks and harms. This calls for putting in place policy provisions, guidelines, and financial allocations that support the provision of comprehensive harm reduction services.

The study indicates how initiation of injecting drug use occurs in over 80% of the PWID in Uganda by the age of 24. As such, harm reduction awareness should be mainstreamed in programs of adolescents and young people.

Due to limited geographically specific data on PWID, similar studies should be conducted for other major towns of Uganda and should include biomarkers for HIV and viral hepatitis testing.

## Data Availability

The datasets used and/or analyzed during this study are available from the corresponding author on reasonable request.

## Abbreviations

FGDs: Focus group discussions
HCV: Hepatitis C virus
HIV: Human immunodeficiency virus
IDI: In-depth interviews
MARPI: Most At Risk Populations Initiative
NSP: Needle and syringe program
PWID: People who inject drugs
TASO: The AIDS Support Organization
